# Rutting vocal display in male impala (*Aepyceros melampus*) and overlap with alarm context

**DOI:** 10.1186/s12983-020-00383-9

**Published:** 2021-01-07

**Authors:** Ilya A. Volodin, Elena V. Volodina, Roland Frey

**Affiliations:** 1 Department of Vertebrate Zoology, Faculty of Biology, Lomonosov Moscow State University, Vorobievy Gory, 12/1, Moscow, 119234 Russia; 2 Department of Behaviour and Behavioural Ecology of Mammals, A.N. Severtsov Institute of Ecology and Evolution, Moscow, Russia; 3 Department of Reproduction Management, Leibniz Institute for Zoo and Wildlife Research, Berlin, Germany

**Keywords:** Acoustic variables, Alarm call, Bout structure, Call sequence, Harem ruminant, Inhalatory and exhalatory vocalization phases, Pant-call, Polygynous mammal, Rutting snort

## Abstract

**Background:**

The rutting vocal display of male impala *Aepyceros melampus* is unique for its complexity among ruminants. This study investigates bouts of rutting calls produced towards potential mates and rival males by free-ranging male impala in Namibia. In particular, a comparison of male rutting and alarm snorts is conducted, inspired by earlier findings of mate guarding by using alarm snorts in male topi *Damaliscus lunatus*.

**Results:**

Rutting male impala produced 4–38 (13.5 ± 6.5) rutting calls per bout. We analyzed 201 bouts, containing in total 2709 rutting calls of five types: continuous roars produced within a single exhalation-inhalation cycle; interrupted roars including few exhalation-inhalation cycles; pant-roars distinctive by a pant-phase with rapidly alternating inhalations and exhalations; usual snorts lacking any roar part; and roar-snorts starting with a short roar part. Bouts mostly started and ended with usual snorts. Continuous roars were the shortest roars. The average duration of the exhalatory phase was longest in the continuous roars and shortest in the pant-roars. The average fundamental frequency (49.7–51.4 Hz) did not differ between roar types. Vocal tract length, calculated by using measurements of the first four vocal tract resonances (formants), ranged within 381–382 mm in all roar types. In the studied male impala, rutting snorts within bouts of rutting calls were longer and had higher values of the upper quartile in the call spectra than alarm snorts produced towards potential danger.

**Conclusions:**

Additional inhalations during the emission of the interrupted and pant-roars prolong their duration compared to the continuous roars but do not affect the fundamental frequency or the degree of larynx retraction while roaring. Alarm snorts are separated from one another by large intervals, whereas the intervals between rutting snorts within bouts are short. Sometimes, rutting snorts alternate with roars, whereas alarm snorts do not. Therefore, it is not the acoustic structure of individual snorts but the temporal sequence and the occasional association with another call type that defines snorts as either rutting or alarm snorts. The rutting snorts of male impala may function to attract the attention of receptive females and delay their departure from a male’s harem or territory.

**Supplementary Information:**

The online version contains supplementary material available at 10.1186/s12983-020-00383-9.

## Background

In many polygynous ruminants, harem-holding males produce rutting calls as a prominent part of courtship behaviour [1–7]. Male rutting display attracts potential mating partners [8], affects female ovulation [9, 10] and deters rival males [11–13]. As in many ruminants, vocalization is a remarkable part of the rutting display in territorial rutting male impala *Aepyceros melampus* [14–18]. The rutting vocal display of male impala comprises bouts of roars and snorts [18].

Acoustic traits of rutting calls in ruminants indicate male quality [1, 19, 20], such as a caller’s body size [4, 5, 8, 13, 20–22], age [4, 5, 23], physical condition [24–26], emotional arousal [27, 28] and dominance [20, 22, 29, 30]. Receptive females are responsive to the traits correlating with large male body size, e.g. the lowered vocal tract resonance frequencies (i.e. formants) of rutting calls as a consequence of longer vocal tracts in larger males [8, 21, 31, 32]. However, see [33] for alternative results.

Sexual selection for rutting calls with low formants may result in a morphological specialization of the male vocal apparatus, including a retractable larynx or an extensible nose. The retractable larynx elongates the vocal tract caudally towards the sternum [34–36], whereas an extensible nose elongates the vocal tract rostrally [7, 37]. Lowered formants as acoustic correlates of an elongated vocal tract during the emission of male rutting calls have been considered as an adaptation for exaggerating apparent body size [34]. Lowered formants due to retraction of the larynx were found in rutting male goitred gazelle *Gazella subgutturosa* [36, 38], red deer *Cervus elaphus* [4, 5, 34, 39], fallow deer *Dama dama* [35] and impala [18].

Another acoustic trait of male quality, a low fundamental frequency (f0 below 50 Hz), i.e. the rate of vocal fold vibration, may be effective for deterring male rivals in polygynous ruminants, such as in fallow deer [13]. In contrast, females of red and sika deer *Cervus nippon* appear to react indifferently towards a low fundamental frequency [31, 40]. However, a correlation between a low f0 and male reproductive success has not yet been documented for polygynous ruminants.

In some ruminants, the male larynx is noticeably enlarged [36, 38, 41]. This enlargement, as in Mongolian gazelle *Procapra gutturosa* [42–44], fallow deer [22, 45] and goitred gazelle [36, 41], may result from sexual selection for a visual signal of high testosterone levels in harem-holding males [36, 41]. In male goitred gazelle, the enlargement of the larynx entails a respective enlargement of the vocal folds, producing rutting roars with an f0 of 23 Hz [38, 41]. The larynx of male impala is not noticeably enlarged, but the vocal folds within the larynx are strongly enlarged and modified and are capable of producing rutting roars with an f0 of 50 Hz [18].

A particularly remarkable trait of male impala rutting vocal display is pant-roaring with a rapid alternation of inhalatory and exhalatory vocalization phases [14–16, 18]. Pant-calls are also reported for two species of marsupials [46–48], two species of rhinos [49–51] and three species of primates [52–55]. Potentially, and in addition to low fundamental and formant frequencies, the rapid alternation of inhalatory and exhalatory phases in male impala rutting calls may function as a further acoustic trait of male quality in harem-holding mammals. However, this function has not been investigated yet. Detailed analysis of the pant-roars in male impala is necessary to provide a basis for future playback studies investigating the potential role of pant-roars as indicators of male quality.

Male impala bouts of rutting calls include three types of roars, differing by the underlying breathing mode [18]. The first type is the continuous roar, with a single exhalatory-inhalatory cycle, the second type is the interrupted roar with few interspersed inhalations and the third type is the pant-roar including a part with a rapid alternation of exhalatory and inhalatory phases [18]. Therefore, male impala may serve as a convenient model for investigating the effects of a panting mode of vocal production on the acoustic traits. Although the different types of roars were already identified in a preceding study [18], the acoustic features of these calls have not yet been investigated in detail and the boundaries between these call types have not yet been established.

In addition to the roars, male impala produce snorts within bouts of rutting calls [18]. Similarly sounding snorts can also be produced when they spot a potential danger [56]. This context-sharing of snort vocalization is reminiscent of the situation in male topi antelope *Damaliscus lunatus*, which produce snorts in both rutting and alarm contexts [57]. In topi, the rutting and alarm snorts are acoustically identical and are equally effective for attracting the attention of receptive females [57]. For male impala, similarity or difference between the rutting and alarm snorts has not yet been demonstrated. The use of snorts in different contexts is interesting though as a potential further example of mate guarding via a sensory exploitation mechanism.

The aim of this study was to investigate the complex rutting vocal display and its overlap with alarm calls in free-ranging male impala *Aepyceros melampus* in Namibia. We analyse in detail the complex structure of male impala bouts of rutting calls. We compare the acoustics of different call types within bouts and estimate a potential influence of additional short inhalations and the panting mode of vocal production on the acoustics of the rutting roars. In addition, we compare the acoustic structure of snorts between rutting and alarm contexts.

## Material and methods

### Ethics statement

The data collection for this study was conducted at the Okambara Elephant Ranch, Namibia, with permission of the owner Christian Schmitt. All research procedures of this study (video and audio recordings) were purely observational. The disturbance of animals during data collection was kept at a minimum. No one single animal suffered due to data collection. All study animals were in the property of the ranch. Common impala are not endangered in Namibia. During data collection, we adhered to the ‘Guidelines for the treatment of animals in behavioural research and teaching’ [58], to the laws on animal welfare for scientific research of Namibia, Germany and the Russian Federation, where the research was conducted, and to the guidelines of research protocol # 2011–36 approved by the Committee of Bio-ethics of Lomonosov Moscow State University.

### Study site, subjects and dates

Audio recordings of the rutting calls and the alarm calls of male common impala (*Aepyceros melampus melampus*) were collected at the fenced 15,000-ha Okambara Elephant Ranch (22.68 S, 18.16 E), located about 130 km east of Windhoek, Namibia, during the highest rutting activity from the 1st to 28th of May 2015. The Okambara Elephant Ranch is a native habitat with approximately 60% bush cover and open areas around artificial watering places, where introduced free-ranging adult male impala are subjected to irregular selective legal hunting during the rut. This population originated in 1994 from about 100 individuals of common impala released on the Okambara Elephant Ranch. During data collection, the entire population of impala on the ranch amounted to approximately 800 individuals [18].

In addition to impala, other large non-carnivorous animal species living at the Okambara Elephant Ranch were kudu *Tragelaphus strepsiceros*, eland *Taurotragus oryx*, waterbuck *Kobus ellipsiprymnus*, blue wildebeest *Connochaetes taurinus*, zebra *Equus burchelli* and *E. zebra*, warthog *Phacochoerus aethiopicus*, giraffe *Giraffa camelopardalis*, African elephant *Loxodonta africana*, and Southern white rhino *Ceratotherium simum*. Carnivorous species living at the Okambara Elephant Ranch were black-backed jackal *Canis mesomelas*, cheetah *Acinonyx jubatus,* leopard *Panthera pardus* and brown hyena *Hyaena brunnea*. The lion, a carnivorous species most strongly affecting impala reproductive behaviour [16], was lacking in the study area. Chacma baboon *Papio ursinus* was the only large primate occurring in the study area.

### Audio recording

Male impala bouts of rutting calls were recorded automatically, whereas male impala series of alarm snorts, produced toward potential danger (human researchers) were recorded manually, with hand-held microphones. For the automated audio recordings (sampling rate 22,050 Hz, 16-bit amplitude resolution, stereo), we used four Song Meter SM2+ devices (Wildlife Acoustics Inc., Maynard, MA, USA). Each device was equipped with two external SMX-II omnidirectional accessory microphones (flat frequency response: 20–20,000 Hz), fixed horizontally at 180° to each other. The devices were set at maximum sensitivity and potentially recorded male impala rutting calls within 100 m around the device in places of most active rut, beforehand identified by the presence of multiple fresh impala tracks and feces. One device was placed on the ground within a large wire-mesh cage protecting it from damage by baboons; the remaining three devices were mounted on trees at a height of 2–2.5 m and protected against baboons by thorn bush branches.

The automated audio recordings were set to 9 min recording, interrupted by 1 min pause (the minimum possible pause for this equipment), from 14:00 to 10:00 of the next day, providing 120 audio wav-files of 9 min length each per device for each 24 h period. Each device was checked every 2–3 days during daytime for replacing the cards and batteries and either left on the place for further recordings or transferred to another site for covering a larger area and recording as many rutting males as possible. In total, 11,030 9-min wav-files (1655 h of recording time) were automatically collected in 9 different recording sites at distances of 0.5–12 km from each other between the 1st and the 28th of May 2015.

The manual audio recordings (sampling rate 48,000 Hz, 16-bit amplitude resolution, mono, distance to animals 10–100 m) were collected using two solid state recorders Marantz PMD-660 (D&M Professional, Kanagawa, Japan) with Sennheiser K6-ME66 cardioid electret condenser microphones (Sennheiser electronic, Wedemark, Germany). In total, we manually collected 207 wav-files of 1–11-min(s) duration (about 8 h of recording time) between the 1st and 28th of May 2015.

### Call samples

Bouts of rutting calls could be easily identified within automated recordings because of the large intervals between successive bouts: the intercall intervals within bouts (ranging from 0.1 s to 5 s) were more than 10 times shorter than the intervals between bouts. Bouts of male impala were determined as compact groups of rutting calls [18] followed by intercall intervals not exceeding 5 s, to formally separate them from the occasionally occurring single roars or snorts.

From the automated recordings, we selected 201 bouts of rutting calls from 7 recording sites for detailed acoustic analyses (Additional file 1: Table S1). Two recording sites did not provide high quality calls appropriate for analysis. From the manual recordings, we selected 38 series of alarm snorts (one alarm snort per series) for detailed acoustic analyses and for comparison with the rutting snorts from the bouts of rutting calls. Call samples for acoustic analyses were created using Avisoft SASLab Pro software (Avisoft Bioacoustics, Berlin, Germany). Before the analyses, the audio files were downsampled to 22,050 Hz for better frequency resolution, converted from stereo to mono mode by screening both channels and selecting the channel with best signal-to-noise ratios, and then high-pass filtered at 50 Hz for partially filtering out the background noise. The filtering did not affect the calculated values of the fundamental frequency f0, as the f0-related variables were evaluated via period of f0 (see below). We checked the automated recordings of rutting calls and selected 201 high-quality bouts with high signal-to-noise ratios, not disrupted by wind or overlapped by calls of other animals. To decrease potential pseudoreplication by repeatedly taking bouts of the same individual, the bouts were selected evenly over the entire 28-day recording period and originated from seven recording sites, separated from each other by distances of 0.5–12 km (Additional file 1: Table S1).

### Call analyses

**Fig. 1:**
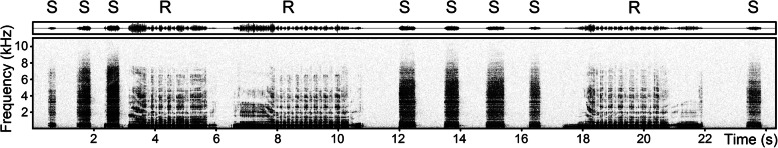
Spectrogram of the bout of male impala rutting calls. (R) roars, (S) snorts. The spectrogram was created with a Hamming window; 22,050 Hz sampling rate; FFT 1024 points; frame 50%; and overlap 87.5%. The audio file of these calls is available as Additional file 2: Audio S2

The 201 bouts of rutting calls comprised two snort types (usual snorts and roar-snorts) and three roar types (continuous roars, interrupted roars and pant-roars) (see Fig. 1 for spectrograms and Results section for description of the call types). For each bout, we measured bout duration and calculated the total number of calls per bout, the number of snorts per bout and the number of roars per bout. For each of the 2709 calls in the 201 bouts, we measured call duration on the screen with the standard marker cursor in the spectrogram window (22,050 Hz sampling rate, Hamming window, FFT 1024 points, frame 50%, overlap 93.75%) by using Avisoft SASLab Pro and the interval to the next call in the bout. In each bout, we calculated the mean intercall interval. For each bout, we calculated time percentage spent vocalizing as the ratio of the total sum of durations of all calls within bout/bout duration.

**Fig. 2:**
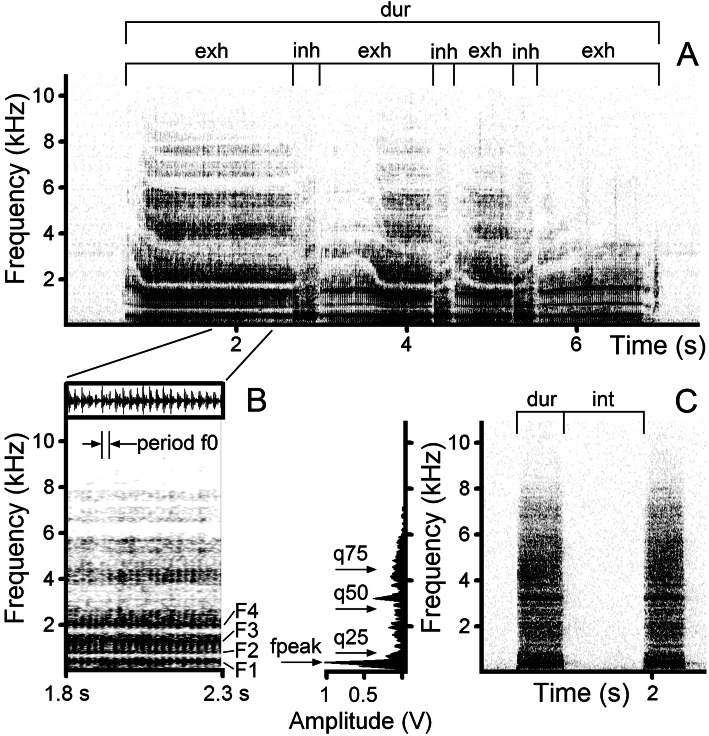
Measured acoustic variables in male impala rutting roars and snorts. **a** Roar variables: call duration (dur), the duration of the exhalatory (exh) and inhalatory (inh) phases. **b** Roar variables on the part from 1.8 s to 2.3 s: fundamental frequency period (period f0), the first four formants (F1-F4). **c** Snort variables: call duration (dur), peak frequency (fpeak), the interval to the next call within the bout (int), the lower (q25), medium (q50), and upper (q75) quartiles, covering respectively 25, 50 and 75% of the energy of the call spectrum

Within bouts, calls separated by intervals of 0.1 s or more were treated as separate calls. This interval was selected as appropriate for separating calls within a bout after checking several hundreds of bouts. From the 201 bouts, we selected for acoustic measurements one roar per bout, in a balanced proportion to the occurrence of the three roar types in the bouts. In total, we measured the acoustic variables in 35 continuous roars, 92 interrupted roars, and 74 pant-roars. In each roar, we measured the duration (dur), the fundamental frequency period (period f0) and the first four formants (F1-F4) (Fig. 2b). The duration and the period f0 (the distance from a previous pulse to the following pulse) were measured from the screen with the standard marker cursor in the main window of Avisoft, displaying the spectrogram and the waveform following [59, 60]. Then we calculated the mean f0 of the exhalatory phase of each roar as the inversed value of the mean period f0 of the roar (Fig. 2b) following [22, 45, 59, 60]. We used the following settings: Hamming window, FFT 512, frame 100%; frequency resolution of the spectrographic analysis was 43 Hz, time resolution varied between 0.3–0.5 ms, depending on call duration.

For each roar (201 roars in total), we also measured the duration of each exhalatory (exh) and inhalatory (inh) phase from the screen with the standard marker cursor in the main window of Avisoft (Fig. 2a). For each roar, we then calculated the number of exhalation-inhalation cycles, the average duration of the exhalatory and inhalatory phases and the percentages of time spent exhaling and inhaling. In pant-roars, we additionally noted the panting part (the part of a pant-roar with rapidly alternating exhalations and inhalations) and calculated the same acoustic variables that were measured for the entire pant-roar for this part separately.

The four first formants (F1, F2, F3 and F4) were tracked with Praat in the roar portion with the lowest formants (Fig. 2b). Formants were measured within roar parts with nearly horizontal formants and their positions were verified by superposition on the narrowband spectrogram. Point values of formant tracks were extracted, exported to Excel (Microsoft Corp., Redmond, WA, USA) and the values of each formant for a given roar were calculated as the average values from the point values. The LPC-settings were Burg analysis, window length 0.04 s, time step 0.01 s, maximum number of formants 4. The upper limit of frequency range of 1800–2200 Hz was selected based on the estimated length of the maximally extended vocal tract of around 400 mm estimated by anatomy-based reconstruction [18].

We calculated the formant dispersion (dF, the mean distance between neighboring formants) as dF = (F4-F1)/3 for each roar, by applying the model of a straight uniform tube closed at one end, following [61]. Then the lengths of the extended vocal tract were calculated by the equation: vocal tract length = C/2dF, where C is the speed of sound in air, approximated as 350 ms ^− 1^ [4, 34]. The formula used to calculate formant dispersion provides only a rough estimation, as the vocal tract of impala is non-uniform [18]. However, calculations of extended vocal tract lengths by using the formant dispersion in impala roars obtained by this method [61] provided values close to those based on anatomical dissection and video single frame pair analysis of vocal tract elongation in impala [18] and in Pannonian red deer *Cervus elaphus hippelaphus* [62]. Furthermore, this method enables calculating dF for each roar and to compare samples between the three types of roars.

To compare the acoustics of the rutting and alarm snorts, we used two different samples of rutting snorts: a sample of 77 usual snorts (one per bout) and a sample of 66 roar-snorts (one per bout). A sample of alarm snorts was selected from the manual recordings of 38 series of alarm snorts (38 alarm snorts, one per series), produced by male impala toward potential danger (researcher). In the usual snorts, roar-snorts and alarm snorts, we measured with Avisoft the duration (dur) in the same way as in the roars and the interval (int) to the next call within a bout or series (Fig. 2c). In addition, we measured the peak frequency (fpeak, the value of the frequency of maximum amplitude), and the lower q25, medium q50, and upper q75 quartiles, covering respectively 25, 50 and 75% of the energy of the call spectrum in the entire-call power spectrum (Fig. 2c). All measurements were exported automatically to Microsoft Excel.

### Statistics

Statistical analyses were conducted using STATISTICA v.8.0 (StatSoft, Tulsa, OK, USA). Means are given as mean ± *SD*, all tests were two-tailed, and differences were considered significant whenever *p* < 0.05. Distributions of 60 measured parameter values of 79 distributions did not depart from normality (Kolmogorov-Smirnov test, *p* > 0.05). As ANOVA is relatively robust to departures from normality [63], this was not an obstacle to the application of the parametric tests.

We used a GLM (General Linear Model) with call type as a fixed factor and recording site as a random factor with Tukey HSD test to compare the acoustics between different types of roars and between different types of snorts. We used a repeated measures ANOVA to compare the values of acoustic variables of the entire pant-roars and of the pant parts of the same roars. We also used a repeated measures ANOVA to compare the durations of exhalations and inhalations within particular exhalatory-inhalatory cycles in the pant parts of the pant-roars.

## Results

We found that successive bouts were separated by intervals of at least a few minutes, because during the 9 min of the automatically recorded wav-file we could see either one bout of rutting calls per file or two bouts, one at the beginning and one at the end of the file. Male impala bouts of rutting calls (*n* = 201 bouts) contained from 4 to 38 (13.5 ± 6.5) calls per bout. Bout duration ranged from 5.4 to 113.2 s (20.6 ± 13.7 s), the mean intercall interval within bouts was 0.56 ± 0.17 s, the average time percentage spent vocalizing within bouts (*n* = 201 bouts) was 64.8 ± 9.6%.

**Fig. 3:**
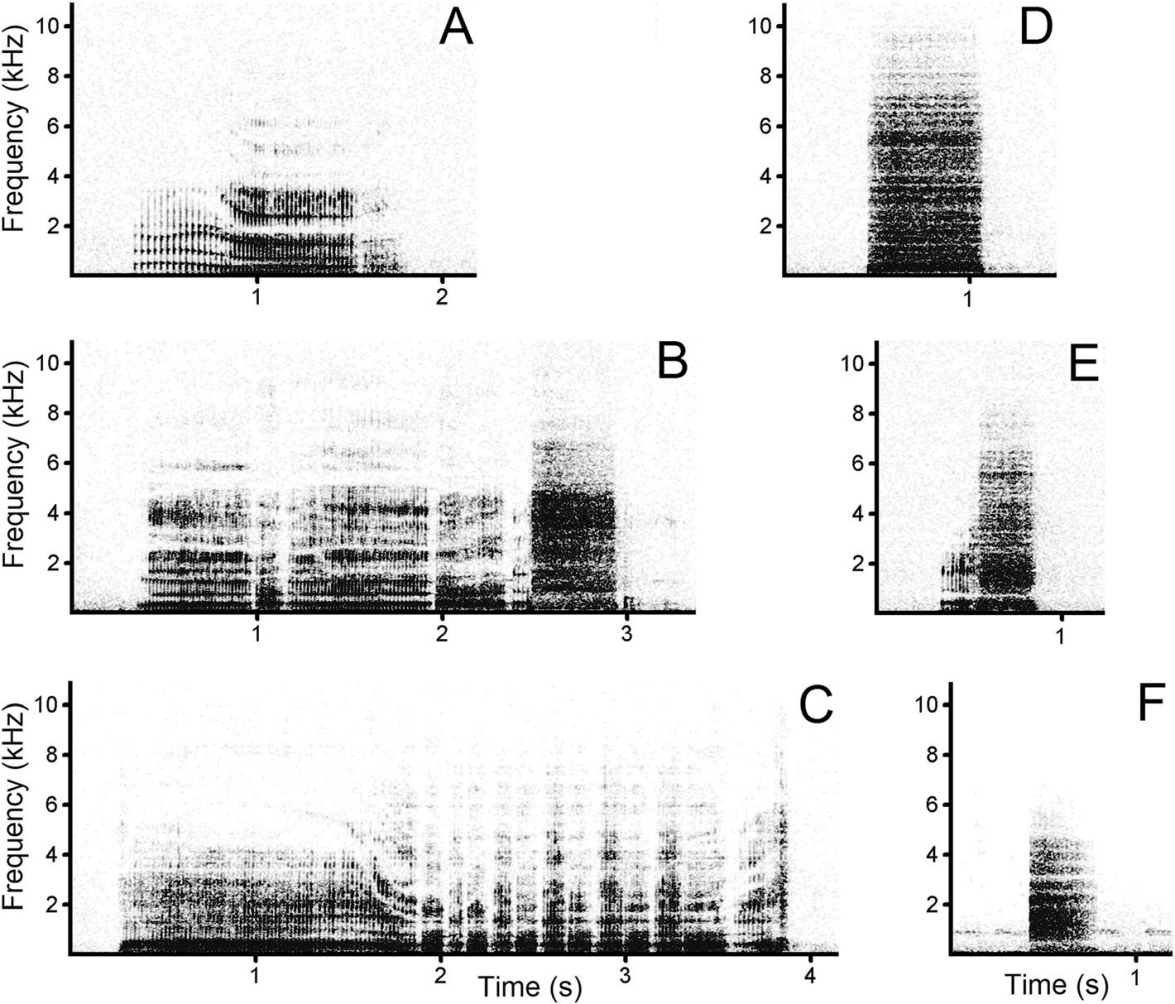
Spectrograms illustrating male impala rutting calls and alarm calls. **a** continuous roar, ending with an inhalation, **b** interrupted roar, ending with a snort without pause, **c** pant-roar, **d** usual snort, **e** roar-snort, **f** alarm snort. Male impala rutting calls are emitted in bouts toward conspecific females and rival males and alarm calls are emitted in series with irregular intervals toward potential danger (researcher). The spectrogram was created with a Hamming window; 22,050 Hz sampling rate; FFT 1024 points; frame 50%; and overlap 93.75%. The audio file of these calls is available as Additional file 3: Audio S3

Bouts of rutting calls could include three types of roars and two types of snorts (Fig. 3). The roars were broadband low-frequency tonal calls with visible pulses of fundamental frequency; snorts were explosive noisy calls without visible fundamental frequency. We identified three types of roars according to differences of the exhalatory-inhalatory cycles during roar production. The continuous roars comprised only a single exhalatory-inhalatory cycle. The interrupted roars comprised from two up to a few exhalatory-inhalatory cycles but lacked pant parts. The pant-roars comprised from two to many exhalatory-inhalatory cycles and obligatorily included a pant part with rapidly alternating exhalations and inhalations (Fig. 3). All male impala snorts were produced at exhalation. Within bouts of rutting calls, we identified two types of snorts: usual snorts (lacking a roar part) and roar-snorts (starting with a roar part shorter than the snort part) (Fig. 3).

Male impala alarm calls comprised exclusively snorts (alarm snorts) produced at exhalation. The alarm snorts were produced by males when they suddenly spotted a researcher at some distance. Males tried to increase the distance from the standing researcher and did not flee but “mobbed” the researcher from a distance, by producing prolonged series of alarm snorts lasting up to 10 min, in which the individual alarm snorts were separated by large irregular intervals (Fig. 3).

The 2709 rutting calls within 201 bouts comprised 6.9% continuous roars, 9.8% interrupted roars, 10.0% pant-roars, 67.3% usual snorts and 6.0% roar-snorts (Table 1). Bouts mostly started with a snort (86.1% of the bouts) and ended with a snort (92.1% of the bouts). Bouts starting with a snort included more calls per bout than bouts starting with a roar (13.9 ± 6.5 and 10.9 ± 1.1 calls respectively, *F*_1,199_ = 5.08, *p* = 0.03). The starting call type did not affect bout duration (*F*_1,199_ = 3.41, *p* = 0.07), the mean intercall interval (*F*_1,199_ = 0.32, *p* = 0.57) and the time percentage spent vocalizing (*F*_1,199_ = 0.54, *p* = 0.46).

**Table 1:** Occurrence of the five call types in male impala bouts of rutting calls

Types of rutting calls	*n* calls	*n* first calls in bouts	*n* last calls in bouts	*n* bouts with this call type	*n* calls/bout mean ± *SD* (min-max)
Continuous roar	188 (6.9%)	2 (1.0%)	3 (1.5%)	81 (40.3%)	2.3 ± 2.1 (1–11)
Interrupted roar	266 (9.8%)	5 (2.5%)	3 (1.5%)	134 (66.7%)	2.0 ± 1.3 (1–12)
Pant-roar	270 (10.0%)	21 (10.4%)	4 (2.0%)	104 (51.7%)	2.6 ± 1.8 (1–11)
Usual snort	1822 (67.3%)	173 (86.1%)	186 (92.5%)	69 (34.3%)	9.1 ± 5.0 (2–25)
Roar-snort	163 (6.0%)	0	5 (2.5%)	201 (100%)	2.4 ± 0.2 (1–9)
All roars	724 (26.7%)	28 (13.9%)	10 (5.0%)	201 (100%)	3.6 ± 2.9 (1–21)
All snorts	1985 (73.3%)	173 (86.1%)	191 (95.0%)	201 (100%)	9.9 ± 5.1 (2–27)
Total	2709	201	201	201	13.5 ± 6.5 (4–38)

All bouts contained both snorts and roars, from 2 to 27 (9.9 ± 5.1) snorts and from 1 to 21 (3.6 ± 2.9) roars per bout (Table 1). Only the usual snorts occurred in 100% of the bouts. Roars comprised 26.8 ± 12.3% of calls within bouts (Table 1). Of the 201 bouts, 101 included one type of roars, 82 included two types of roars and 18 included all three types of roars. Of the 724 roars in the 201 bouts, 230 (32%) roars ended with a snort without pause (Fig. 3b). Of the 230 roars ending with a snort, 26% were continuous roars, 35% were interrupted roars and 33% were pant-roars.

Acoustic comparison of 35 continuous roars, 92 interrupted roars and 74 pant-roars did not reveal any differences in the mean f0 (ranging from 49.7 to 51.4 Hz) or the values of the first F1, second F2 and fourth F4 formants (Table 2). Call duration was shortest in the continuous roars. The third formant F3 was higher in the interrupted than in the continuous roars; it was intermediate in pant-roars and did not differ significantly from other roars (Table 2). The maximally extended vocal tract length during roar-synchronous retraction of the larynx, calculated on the basis of formant dispersion, ranged from 381 to 382 mm and did not differ between the three types of roars (Table 2). The factor “recording site” significantly affected all acoustic variables of the rutting roars (Table 2).

**Table 2:** Values (mean ± *SD*) of the acoustic variables of the three types of male impala rutting roars

Roar variable	All roars *n* = 201	Continuous roars *n* = 35	Interrupted roars *n* = 92	Pant-roars *n* = 74	GLM roar type effect	GLM recording site effect
Dur (s)	2.89 ± 1.66	1.65 ± 0.47 ^a^	3.04 ± 1.79 ^b^	3.28 ± 1.59 ^b^	***F***_**2,192**_ **= 14.2,** ***p*** **< 0.001**	***F***_**6,192**_ **= 10.4,** ***p*** **< 0.001**
f0mean (Hz)	50.5 ± 4.8	49.7 ± 3.4	50.1 ± 3.6	51.4 ± 6.3	*F*_2,192_ = 0.43, *p* = 0.65	***F***_**6,192**_ **= 5.21,** ***p*** **< 0.001**
F1 (Hz)	349 ± 28	345 ± 31	353 ± 27	347 ± 27	*F*_2,192_ = 0.99, *p* = 0.38	***F***_**6,192**_ **= 3.19,** ***p*** **= 0.005**
F2 (Hz)	745 ± 53	746 ± 59	743 ± 54	747 ± 49	*F*_2,192_ = 0.39, *p* = 0.68	***F***_**6,192**_ **= 3.91,** ***p*** **= 0.001**
F3 (Hz)	1270 ± 66	1245 ± 61 ^a^	1284 ± 66 ^b^	1264 ± 66 ^a, b^	***F***_**2,192**_ **= 3.18,** ***p =*** **0.04**	***F***_**6,192**_ **= 9.20,** ***p*** **< 0.001**
F4 (Hz)	1741 ± 134	1738 ± 154	1747 ± 136	1734 ± 121	*F*_2,192_ = 0.59, *p =* 0.56	***F***_**6,192**_ **= 10.5,** ***p*** **< 0.001**
dF (Hz)	464 ± 44	464 ± 51	464 ± 45	462 ± 38	*F*_2,192_ = 0.32, *p* = 0.73	***F***_**2,198**_ **= 8.46,** ***p*** **< 0.001**
vtl (mm)	381 ± 39	382 ± 45	381 ± 41	381 ± 33	*F*_2,192_ = 0.56, *p* = 0.57	***F***_**6,192**_ **= 7.82,** ***p*** **< 0.001**
*n* exhalation-inhalation cycles	4.67 ± 3.50	1.00 ± 0.00 ^a^	3.63 ± 1.69 ^b^	7.69 ± 3.58 ^c^	***F***_**2,192**_ **= 109.1,** ***p*** **< 0.001**	***F***_**6,192**_ **= 11.6,** ***p*** **< 0.001**
one exhalatory phase duration (s)	0.64 ± 0.46	1.40 ± 0.43 ^a^	0.63 ± 0.27 ^b^	0.30 ± 0.10 ^c^	***F***_**2,192**_ **= 246.2,** ***p*** **< 0.001**	***F***_**6,192**_ **= 8.32,** ***p*** **< 0.001**
one inhalatory phase duration (s) ^a^	0.21 ± 0.11	0.31 ± 0.13 ^a^	0.24 ± 0.11 ^b^	0.15 ± 0.03 ^c^	***F***_**2,179**_ **= 36.5,** ***p*** **< 0.001**	***F***_**6,179**_ **= 6.93,** ***p*** **< 0.001**
time spent exhaling (%)	75.6 ± 10.8	87.3 ± 11.4 ^a^	76.8 ± 8.1 ^b^	68.6 ± 7.8 ^c^	***F***_**2,192**_ **= 52.5,** ***p*** **< 0.001**	***F***_**6,192**_ **= 7.15,** ***p*** **< 0.001**
time spent inhaling (%)	24.4 ± 10.8	12.7 ± 11.4 ^a^	23.2 ± 8.1 ^b^	31.4 ± 7.8 ^c^	***F***_**2,192**_ **= 52.5,** ***p*** **< 0.001**	***F***_**2,198**_ **= 7.15,** ***p*** **< 0.001**

Designations: *Dur* call duration, *f0mean* the mean fundamental frequency, *F1, F2, F3, F4* the four first formants, *dF* the formant dispersion, *vtl* the vocal tract length calculated on the basis of formant dispersion for the maximally retracted larynx position during roar emission, *n* exhalation-inhalation cycles = number of exhalation-inhalation cycles, *GLM* GLM results, significant differences are given in bold. The same superscripts indicate that the values did not differ significantly (Tukey HSD test)

^a^*n* = 22 for the continuous roars

All roars always started with an exhalation, but only 160 of the 201 roars (79.6%) ended with an exhalation. The remaining 41 roars (20.4%) ended with an inhalation, clearly visible in the spectrograms (Fig. 3a). Mostly, the continuous roars, 17 of 35 roars (48.6%), were ending with an inhalation. Values of the acoustic variables related to exhalation-inhalation cycles, differed strongly among the three types of roars (Table 2). In the continuous roars, the number of exhalation-inhalation cycles was always equal to 1, whereas in the pant-roars, the maximal number of exhalation-inhalation cycles was 21 (7.69 cycles on average). In the continuous roars, the average duration of the exhalatory phase exceeded that in the interrupted roars twice and exceeded that in the pant-roars four times. In the continuous roars, the inhalatory phase was the longest among the three types of roars. Time spent exhaling for the duration of a roar progressively decreased from continuous roars to pant-roars, whereas the time spent inhaling progressively increased from continuous roars to pant-roars (Table 2).

In the pant parts of the pant-roars, the number of exhalation-inhalation cycles comprised 4.5 ± 3.1 cycles on average (from 2 to 18 cycles in different pant-roars). Repeated measures ANOVA for comparison between the pant parts and the entire call in the same pant-roars showed that the exhalatory phases of the pant part (0.17 ± 0.05 s) were shorter on average than those of the entire roar (*F*_1,73_ = 173.1, *p* < 0.001). Similarly, the inhalatory phases of the pant part (0.13 ± 0.02 s) were significantly shorter than those of the entire pant-roar (*F*_1,73_ = 76.0, *p* < 0.001).

Consistently, the average total time spent exhaling was shorter during the pant parts of the pant roars (57.3 ± 6.4%) than during the entire pant-roars, whereas the average time spent inhaling was longer during the pant parts of the pant roars (42.7 ± 6.4%) than during the entire pant-roars (*F*_1,73_ = 245.9, *p* < 0.001 for both comparisons). Thus, inhalations and exhalations alternated very rapidly during the pant phases of pant-roars. Time spent exhaling during the pant phases only slightly exceeded time spent inhaling, although the exhalations were significantly longer than the subsequent inhalations within particular exhalation-inhalation cycles (repeated measures ANOVA taking into account the belonging of an exhalation-inhalation cycle to a particular roar, *F*_1,259_ = 556.8, *p* < 0.001).

Comparison of the usual snorts, roar-snorts and alarm snorts revealed that the alarm snorts were the shortest, had the lowest upper quartile and had the longest interval to the next call. The usual snorts and roar-snorts did not differ by all measured variables (Table 3). The range of the alarm snort duration (min-max 0.14–0.63 s) strongly overlapped with duration ranges of the usual snort (0.15–0.91 s) and the roar-snort (0.16–0.86 s). The range of the alarm snort upper quartile q75 (1200–4410 Hz) weakly overlapped with those of the usual snort (2320–5660 Hz) and the roar-snort (1720–5550 Hz). The range of the alarm snort inter-call interval (2.71–52.20 s) did not overlap with those of the usual snort (0.14–1.73 s) and the roar-snort (0.12–1.16 s). The factor “recording site” significantly affected three of the six acoustic variables of snorts (Table 3).

**Table 3:** Values (mean ± *SD*) of acoustic variables of the three types of male impala snorts

Snort variable	All snorts *n* = 181	Usual snorts *n* = 77	Roar-snorts *n* = 66	Alarm snorts *n* = 38	GLM snort type effect	GLM recording site effect
Dur (s)	0.39 ± 0.15	0.42 ± 0.15 ^a^	0.43 ± 0.14 ^a^	0.28 ± 0.09 ^b^	***F***_**2,172**_ **= 15.5,** ***p*** **< 0.001**	*F*_6,172_ = 1.58, *p* = 0.15
fpeak (Hz)	1070 ± 566	1066 ± 598	997 ± 547	1207 ± 517	*F*_2,172_ = 1.64, *p* = 0.18	***F***_**6,172**_ **= 2.55,** ***p*** **= 0.02**
q25 (Hz)	1103 ± 277	1113 ± 275	1050 ± 258	1177 ± 300	*F*_2,172_ = 2.40, *p* = 0.10	***F***_**6,172**_ **= 2.88,** ***p*** **= 0.01**
q50 (Hz)	1995 ± 487	2103 ± 504	1935 ± 428	1879 ± 513	*F*_2,172_ = 2.63, *p* = 0.08	*F*_6,172_ = 1.15, *p* = 0.34
q75 (Hz)	3363 ± 734	3462 ± 581 ^a^	3532 ± 763 ^a^	2870 ± 766 ^b^	***F***_**2,172**_ **= 11.0,** ***p*** **< 0.001**	***F***_**6,172**_ **= 2.31,** ***p*** **= 0.04**
Int (s) ^a^	3.10 ± 7.06	0.56 ± 0.32 ^a^	0.42 ± 0.23 ^a^	13.13 ± 10.79 ^b^	***F***_**2,146**_ **= 80.7,** ***p*** **< 0.001**	*F*_6,146_ = 1.56, *p* = 0.16

Designations: *Dur* call duration, *fpeak* the peak frequency with maximum amplitude, *q25, q50, q75* the lower q25, medium q50, and upper q75 quartiles, covering 25, 50 and 75% of the energy of the call spectrum, *Int* Interval to the next call within bout or series, *GLM* GLM results, significant differences are given in bold. The same superscripts indicate that the values did not differ significantly (Tukey HSD test)

^a^*n* = 58 for the usual snorts, *n* = 65 for the roar-snorts, *n* = 32 for the alarm snorts

## Discussion

### Bouts of rutting calls

This study analysed the rutting calls of male impala, emitted in compact bouts comprising three types of roars and two types of snorts. The bouts were complex displays, containing roars, putatively produced by the vocal folds, and snorts, produced by explosive expirations through the nose, as documented by our video footage. In other ruminants, bouts of rutting calls can also include different call types: roars, growls and grunts in goitred gazelle [36] or harsh and common long and short roars in red deer [4, 39, 62, 64, 65]. However, among the studied species of ruminants, only male impala bouts include both roars and snorts.

Male impala frequently use their bouts of rutting calls to attract potential female mating partners and to defend them against rival males. Therefore, this vocal display is a dominant part of male impala rutting behaviour and might be costly regarding the energy budget [14–16, 18]. Bouts of rutting calls of male impala follow each other with large intervals (of at least a few minutes), indicating that their production could be exhausting for a caller. In addition, roar emission by males involves other potentially exhaustive activities, as retraction of the larynx by maximal contraction of the strap muscles and pronounced inhalatory and exhalatory phases supported by strong intermittent contractions of the abdominal muscles [18]. Apparently, this also applies to the advertising display in male koalas, which also produce their sequences of pant-calls at large intervals and accompanied by pronounced flank movements [66].

Moreover, male impala often emit their bouts of rutting calls while running [18] and, thus, making their production particularly challenging in terms of energy expenditure. Male impala bouts contained many rutting calls (13.5 on average, up to a maximum of 38) and were of long duration (on average 20.6 s). For comparison, bouts of rutting calls in another bovid, the goitred gazelle, also produced while running, contain only 2.67 calls per bout on average at an average bout duration of 1.28 s [36]. In cervids (red deer stags), commonly producing their bouts of rutting roars from a standing posture, the bouts are also shorter and contain less calls on average than in male impala: 2.6 roars at an average bout duration of 1.63 s in Corsican red deer *Cervus elaphus corsicanus* [64], 2.11 roars at an average bout duration of 3.41 s in Iberian red deer *C. e. hispanicus* [39] and 3.18 roars at an average bout duration of 3.73 s in Pannonian red deer *C. e. hippelaphus* [62].

### Roars

The estimated length of the maximally extended vocal tract of male impala did not differ between roar types. Apparently, the additional short inhalations in the interrupted roars and pant-roars did not affect the degree of larynx retraction and vice versa. Similarly, the fundamental and formant frequencies of the roars were not affected by the additional inhalations in the interrupted and pant-roars (except a weak influence on the third formant F3). Male impala retract the larynx down to a mid-neck position [18], i.e. less far than the other ruminant species known to retract the larynx down towards the sternum [34–36, 39, 42]. The fundamental frequency of male impala rutting roars was low (50.5 Hz) and comparable to the low fundamental frequency in the rutting groans of male fallow deer (from 21 to 39 Hz, 28.2 Hz on average) [24, 45] and in the rutting roars of male goitred gazelle (from 17.2 to 27.8 Hz, 22.0 Hz on average) [36]. In fallow deer and goitred gazelle, there is a strong sexual dimorphism of the larynx and vocal folds [41]. In contrast, there is no obvious sexual dimorphism of larynx size in impala and the male larynx is not enlarged. Probably, the low fundamental frequency of impala roars results from the enlargement and unique structure of the massive male vocal folds [18].

### Pant-calls

In male impala, the exhalatory phase displayed clear pulses, evidently resulting from the regular vibration of the sound source (most probably the vocal folds), whereas during the inhalatory phase, the sound was noisy (Fig. 2a). This difference between the exhalatory and inhalatory phases is reminiscent of the koala male advertising calls [66] and the striped possum *Dactylopsila trivirgata* mating calls [48], produced at both phases of respiration, in spite of the distinctive mechanism of sound production in the koala [46, 67]. In contrast, clear pulsation is evident at both exhalatory and inhalatory phases of the purring vocalizations of felids [68, 69], probably because of the involvement of active muscle contractions for producing the exhalatory and inhalatory phases of these calls [70].

In male impala, there is a gradual shortening of both exhalations and inhalations from continuous roars via interrupted roars towards pant-roars and further to the pant parts of the pant-roars. From continuous via interrupted to pant-roars, exhalations and inhalations alternated more and more rapidly, and the time spent inhaling progressively increased. In the pant-parts of the pant-roars, within particular exhalation-inhalation cycles, the inhalations were shorter than the exhalations. In contrast, in the pant-calls of a marsupial species, the striped possum, the exhalations were always shorter than the subsequent inhalations [48].

In male impala, both the pant-roars and the interrupted roars were two times longer than the purely exhalatory continuous roars. Pant-roaring as well as short additional inhalations within the interrupted roars, might therefore promote the production of longer roars. The longer duration of the roars and the corresponding longer duration of the bouts of rutting calls may be important for advertising male quality. Both the extra inhalations and the longer calls might be challenging for the male respiratory system and the entire male physiology and energy budget. In koala, pant-calling in advertising/pair-bonding contexts may be similarly challenging to both sexes [46, 48].

Pant-calls appear to be an adaptation for producing impressive vocal displays as they have an increased audibility [49, 50, 71]. However, pant-calls may function differently across taxa. Whereas in impala only males produce pant-roars and only in a rutting context, both sexes of striped possums and koalas produce pant-calls during pair formation, probably not only for mate attraction but also as an agonistic or territorial display [47, 48]. In male and female koalas, pant-calls announce individual identity, age and sex [47, 66]. In male and female southern white rhinoceros *Ceratotherium simum*, pant-calls announce individual identity and age in different social contexts [51, 72]. In female baboons, pant-calls represent copulation calls [52–55], announcing individual identity [53] and proximity to ovulation [54]. In male chimpanzee, pant hoots announce male quality [73, 74], testosterone levels [75], individual identity [76] and social context [77]. In hairy armadillo *Chaetophractus vellerosus*, pant-calls function as distress vocalizations [78]. Loud calls at both respiratory phases were also reported in donkeys *Equus asinus* [79], but the function of these calls has not yet been clarified. Ultimately, pant-calls occur in many species but they are rarely emitted in all taxa. The contexts in which pant-calls occur are diverse, ranging from aggressive to peaceful, and their functional meaning is poorly understood.

### Origin of pant-calling from thermoregulatory behaviour

From an evolutionary perspective, respiratory panting evolved as an adaptation for thermoregulation to avoid overheating by evaporative cooling [80–82]. In the course of further evolution, panting, by an expansion of its function, could have acquired an additional or, by a change of function [83], even a sole acoustic role, e.g. for enhancing the impressiveness of vocalizations, as in male impala (this study), rhinos [51, 72], koalas [46], striped possums [48] and lions *Panthera leo* [84]. Indirect evidence for this comes from the fact that pant-calling occurs only in animals living in hot climates, as koalas [85], striped possums [86], rhinos [51, 72], chimpanzee [73], lions [87] and impala [16].

Impala experience thermal stress between 35 °C and 50 °C of ambient temperature and their breathing rate at such temperatures can exceed 200 cycles per minute [88]. Therefore, panting might be adaptive for evaporative cooling in male impala. As a consequence, pant-roaring in rutting male impala could have evolved as a mechanism to avoid overheating during their exaustive rutting vocal display. The ability of males to use pant-roars for effective evaporative cooling may even be an indirect trait of male quality. Possibly, those males which are capable of producing long rutting vocal displays without overheating because of their efficient thermoregulation are more attractive for females.

Another possible adaptation against overheating in male impala is tongue protrusion during the rutting vocal display. This behaviour might also involve a thermoregulatory function besides its potential role as a visual display [18]. Thermoregulatory and advertising roles of tongue protrusion during the rutting vocal display have already been discussed for Iberian red deer *Cervus elaphus hispanicus* [39]. Often, pure thermoregulatory panting is accompanied by tongue protrusion both in carnivores (e.g. domestic dogs *Canis familiaris*) and in ruminants (e.g. reindeer *Rangifer tarandus*) [80–82, 89, 90] indicating the thermoregulatory role of tongue protrusion. However, the rutting period of goitred gazelle occurs at relatively low temperatures, and a wide opening of the mouth with tongue protrusion has also been documented for the vocal display of male goitred gazelle [36, 91]. Presumably, the advertising role of tongue protrusion prevails over its thermoregulatory role in male goitred gazelle.

Nevertheless, tongue protrusion in male impala during pant-roar emission might be a behavioural relict of an ancestral purely thermoregulatory panting [81, 82]. Perhaps, tongue protrusion has been retained to prevent or restrict the formation of an adequate oropharyngeal seal and, thereby, to facilitate inhalation and exhalation through the mouth, as well as through the nose, during pant-roar emission. The observed roar-synchronous wide opening of the nostrils would support this assumption. During deep open mouth panting in dogs and reindeer, the airflow is bidirectional in both nose and mouth but only a small fraction of the air is routed through the nose because of its higher resistance [92]. Consequently, the bulk of the air passes in and out through the oropharynx [80, 81]. From a bioacoustical perspective, this would suggest an involvement of the nasal vocal tract in pant-roar production although to a much lesser degree than of the oral vocal tract.

An involvement of the nasal vocal tract in production of the rutting groans has been suggested for male fallow deer [93]. In male impala, the values of the oral vocal tract length calculated on the basis of formants and the values obtained by anatomical and video analyses are very close (they practically coincide) [18]. This contradicts the involvement of the nasal vocal tract in impala roaring. In red deer, some rutting calls start nasally and then shift to purely oral calling. This is clearly visible in spectrograms, as the nasal part is always much fainter than the oral part [26]. However, the spectrograms of male impala roars do not reveal any nasal parts. This suggests that nasalization during roaring in male impala is negligibly small or lacking. Accordingly, the roar-synchronous opening of the nostrils might be used for increasing the inhalation efficiency by allowing oronasal inhalations.

### Rutting and alarm snorts

We found that rutting snorts of both types (usual snorts and roar-snorts) did not differ from each other in any acoustic variable, whereas the alarm snorts were shorter and had a lower upper quartile, i.e. their sound energy was stronger concentrated in the middle frequency range. However, there was substantial overlap regarding duration and upper quartile between samples of rutting and alarm snorts. Hence, recipient animals can hardly discriminate between rutting and alarm snorts by judging from the hearing of a single snort. This is especially important in natural habitats, where the call duration can be distorted by echo, and distribution of sound energy depends on the distance to a caller [94–96]. The prominent difference between alarm snorts of the series and rutting snorts of the bouts arises from the specific sequence combination pattern of snorts. Alarm snorts are separated from one another by large intervals, whereas the intervals between rutting snorts within bouts are short. Sometimes, rutting snorts alternate with roars, whereas alarm snorts do not. Therefore, it is not the acoustic structure of individual snorts but the temporal sequence pattern and the association with another call type or not that defines snorts as either rutting or alarm snorts. These results are consistent with findings indicating the importance of call combinations in the communicative systems of mammals, including impala, and birds [97–99].

Playback experiments showed that male impala [99], like female topi [57], respond to any isolated snorts as to alarm calls. In contrast, the combination of snorts and roars provoked an aggressive reaction in male impala [99], whereas in female impala it provoked only an increase in their movements but did not alarm them [100]. Further research is necessary to reveal the precise functions of different call types of male impala and their use in relation to season and climate conditions.

Impala therefore, like topi antelope, display snorts in both an alarm and a rutting context [57]. The rutting snorts of male impala may function to attract the attention of receptive females and delay their departure from a male’s harem or territory. However, in topi, the snorts are the only calls described in the rutting context, whereas male impala produce bouts of roars and snorts during their rutting behaviour.

In many ruminants, snorts are used as alarm calls, for review see [56]. However, among bovids, only in topi [57] and in impala (this study) the alarm snorts are included in the courtship display. In cervids, there are observations of manipulative use of alarm barks by red deer stags for promoting defensive bunching by the hinds and thus to increase the cohesion of a stag’s harem [5].

Aside from ruminants, males of two rodent species (Pallas’s squirrel *Callosciurus erythraeus* and Belding’s ground squirrel *Urocitellus beldingi*) also produce alarm calls in relation to sexual behaviour in precopulatory and postcopulatory contexts [101, 102]. The hypothetical function of these alarm calls is manipulation of females via a sensory exploitation mechanism, allowing the calling males postcopulatory mate guarding to avoid matings with other males and subsequent sperm competition [101, 102]. Aside from mammals, the superb lyrebird, *Menura novaehollandiae* uses alarm calls in a sexual context during mating [103].

The rutting activity of impala depends on climatic conditions. A clearly restricted rutting season with high levels of roaring activity exists only in subtropical zones with seasonal changes of temperature, day length and rainfall, as e.g. in Namibia [18]. In habitats with a tropical climate, as e.g. in Kenya, impala breed continuously and dominant impala males permanently herd females and defend them against rival males, producing roaring activity at a moderate level [104].

## Conclusion

Rutting calls of male impala are produced in bouts comprising three types of roars and two types of snorts. Pant-roars, including multiple short inhalations, represent the longest roars within bouts, whereas the interrupted roars with few inhalations are shorter and the continuous roars without inhalations are the shortest. We therefore conclude that additional inhalations facilitate the production of longer roars. The average fundamental frequency (49.7–51.4 Hz) does not differ between roar types, indicating that additional inhalations do not affect the produced fundamental frequency. Vocal tract length, estimated by using measurements of the first four vocal tract resonances (formants), ranges within 381–382 mm in all roar types, indicating a similar degree of maximal larynx retraction during their emission. In impala, pant-calling with tongue protrusion might have evolved as an adaptation against overheating during their exhausting rutting vocal display in a hot climate. In addition to topi antelope, impala is the second species of ruminants, in which the males are displaying snorts in both an alarm and a rutting context.

## Supplementary Information

**Additional file 1: Table S1.** Bouts of male impala rutting calls included in acoustic analysis per recording site.

**Additional file 2: Audio S2.** Sound file (.wav) of the bout of male impala rutting calls.

**Additional file 3: Audio S3.** Sound file (.wav) of male impala rutting calls emitted in bouts: continuous roar, interrupted roar, pant-roar, usual snort, roar-snort, and alarm snort toward potential danger (researcher).

## Data Availability

The datasets used and/or analyzed during the current study are available from the corresponding authors on reasonable request.

## Abbreviations

dF: The formant dispersion; dur: Call duration
exh: The duration of the exhalatory phase
f0: The fundamental frequency
f0mean: The mean fundamental frequency
F1: The first formant
F2: The second formant
F3: The third formant
F4: The fourth formant
FFT: Fast Fourier Transform
fpeak: The peak frequency with maximum amplitude
inh: The duration of the inhalatory phase
Int: Interval to the next call within bout or series
q25: The lower quartile, covering 25% of the energy of the call spectrum
q50: The medium quartile, covering 50% of the energy of the call spectrum
q75: The upper quartile, covering 75% of the energy of the call spectrum
vtl: The vocal tract length
