# Supplementary material for: Rutting vocal display in male impala (Aepyceros melampus) and overlap with alarm context
Source: Front Zool. 2021 Jan 7;18:2. doi: 10.1186/s12983-020-00383-9 (PMC7792082; doi:10.1186/s12983-020-00383-9)
Supplement: Supplementary file 1 — Additional file 1: Table S1. Bouts of male impala rutting calls included in acoustic analysis per recording site. [file 12983_2020_383_MOESM1_ESM.pdf]

Table S1. The numbers of bouts of male impala rutting calls per recording site that were included in the acoustic analysis.

Recording site ID	SongMeter ID	N bouts	Data start	Data end	N days	Bouts per day
01	V	47	01.05.2015	28.05.2015	27	1.74
19	C	68	04.05.2015	28.05.2015	24	2.83
12	B	56	02.05.2015	17.05.2015	15	3.73
61	B	3	17.05.2015	24.05.2015	7	0.43
57	B	13	24.05.2015	28.05.2015	4	3.25
05	A	0	01.05.2015	04.05.2015		
09	A	0	04.05.2015	10.05.2015		
41	A	1	10.05.2015	12.05.2015	2	0.5
47	A	13	13.05.2015	28.05.2015	14 *	0.93
Total		201			93	
Mean					13.29	1.92
SD					9.66	1.36

Designations: N bouts − number of bouts of male impala rutting calls; Data start − calendar date of the start of the automated acoustic recording at the given recording site; Data end − calendar date of the end of the automated acoustic recording at the given recording site; N days − the number of days (20 hours per day) of acoustic recording at the given recording site; Bouts per day − the number of bouts of rutting calls per day of recording for each recording site; * - 1 day without recording.
